# Structural genome analysis in cultivated potato taxa

**DOI:** 10.1007/s00122-019-03519-6

**Published:** 2019-12-31

**Authors:** Maria Kyriakidou, Sai Reddy Achakkagari, José Héctor Gálvez López, Xinyi Zhu, Chen Yu Tang, Helen H. Tai, Noelle L. Anglin, David Ellis, Martina V. Strömvik

**Affiliations:** 1 Department of Plant Science, McGill University, Sainte-Anne-de-Bellevue, Montreal, QC H9X 3V9 Canada; 2 Fredericton Research and Development Centre, Agriculture and Agri-Food Canada, Fredericton, Canada; 3 International Potato Center, Lima, Peru

## Abstract

**Key message:**

Twelve potato accessions were selected to represent two principal views on potato taxonomy. The genomes were sequenced and analyzed for structural variation (copy number variation) against three published potato genomes.

**Abstract:**

The common potato (*Solanum tuberosum* L.) is an important staple crop with a highly heterozygous and complex tetraploid genome. The other taxa of cultivated potato contain varying ploidy levels (2*X*–5*X*), and structural variations are common in the genomes of these species, likely contributing to the diversification or agronomic traits during domestication. Increased understanding of the genomes and genomic variation will aid in the exploration of novel agronomic traits. Thus, sequencing data from twelve potato landraces, representing the four ploidy levels, were used to identify structural genomic variation compared to the two currently available reference genomes, a double monoploid potato genome and a diploid inbred clone of *S. chacoense*. The results of a copy number variation analysis showed that in the majority of the genomes, while the number of deletions is greater than the number of duplications, the number of duplicated genes is greater than the number of deleted ones. Specific regions in the twelve potato genomes have a high density of CNV events. Further, the auxin-induced SAUR genes (involved in abiotic stress), disease resistance genes and the 2-oxoglutarate/Fe(II)-dependent oxygenase superfamily proteins, among others, had increased copy numbers in these sequenced genomes relative to the references.

**Electronic supplementary material:**

The online version of this article (10.1007/s00122-019-03519-6) contains supplementary material, which is available to authorized users.

## Introduction

Cultivated potato (*Solanum tuberosum* L.) originated in the Andean highlands of southern Peru. Whereas potato was not cultivated in Europe and other parts of the world until the sixteenth century, archeological evidence suggests that the potato has been used for human consumption in Peru for at least 10,000 years (Engel 1970). Since ancient times, potato has been adopted into the human diet and is today the third most important food crop for direct human consumption globally (fao.org).

This worldwide success of potato as a crop is in part due to the tubers being highly nutritious and providing a good source of fiber, minerals, proteins and vitamins C and B6. Important in the adoption of potato as a human food is its wide adaptability to varying environmental conditions and climates—it is grown from the Americas, to Africa, Eurasia and Oceania, and in a broad range of conditions such as differential elevation (Bradeen et al. 2011). However, genetic improvement in existing cultivars is necessary to meet the global food and nutritional demands from a changing climate and the growing human population. The great diversity in potato species and landraces, in particular the South American potato taxa, which contain a rich source of valuable agronomic traits, offers insights into the genetic diversity behind the adaptability of the common cultivated potato. Insights into the genomic variation of the diversity of cultivated potato taxa are crucial to crop improvement to help combat future famines and ensure food security.

A significant amount of baseline work has previously been done to aid the advance of potato genomics (Galvez Lopez et al. 2017). The first publicly available potato reference genome was derived from a doubled monoploid clone of *S. tuberosum* group *Phureja* (DM1-3), which was sequenced and assembled by the Potato Genome Sequencing Consortium (PGSC 2011). The DM1-3 genome assembly consists of 12 pseudomolecules with a total assembly length of ~ 844 Mb. DM1-3 was soon followed by the reference genome of a *S. chacoense* clone, M6 (Leisner et al. 2018). Additionally, a gene expression atlas of 32 developmental and stress conditions of DM1-3 is available (Massa et al. 2011, 2013) as are several studies on transcriptomes (Gálvez et al. 2016; Barandalla et al. 2018; Moon et al. 2018; Fogelman et al. 2019). The availability of the two potato reference genomes, along with expression data, has facilitated genetic profiling of different potato varieties, particularly in the identification of structural variants such as single nucleotide polymorphisms (SNPs) and larger copy number variations (CNVs). A comparison of 12 monoploid and doubled monoploid clones derived from *S. tuberosum* accessions, to the DM1-3 reference genome, showed that great heterogeneity in the genomes and that a large portion of their genomes are affected by CNVs (Hardigan et al. 2016).

Potato genomic studies have revealed that CNVs play a major role in developing or contributing to adaptive traits (Iovene et al. 2013; Hardigan et al. 2016; Hardigan et al. 2017; Pham et al. 2017). This is in agreement with studies in other crop plants, e.g., the response to stress in *Oryza* species (Bai et al. 2016), and disease resistance in maize (Beló et al. 2010), sorghum (Zheng et al. 2011) and soybean (McHale et al. 2012). Furthermore, a SNP analysis of six potato cultivars showed that large allelic variation correlated with preferential allele expression and was significantly associated with evolutionary conserved genes (Pham et al. 2017).

*Solanum commersonii* is a diploid tuber-bearing wild potato species native to Central and South America. It is thought to be the first wild potato collected on a scientific expedition (Hawkes 1990) and is phylogenetically distinct from cultivated potato (*S. tuberosum*) (Rodríguez and Spooner 2009). *S. commersonii* has desirable agricultural traits not commonly found in the cultivated potato, such as resistance to root knot nematode, soft rot and blackleg, bacterial and verticillium wilt, *Potato Virus X*, tobacco etch virus, common scab and late blight as well as frost tolerance and good capacity for cold acclimation (Hanneman and Bamberg 1986; Hawkes 1990; Micheletto et al. 2000). Breeders have overcome the sexual incompatibility of *S. commersonii* and *S. tuberosum* (Johnston et al. 1980), yet, unfortunately, significant new varieties have still to be released (Cardi et al. 1993; Bamberg 1994; Carputo et al. 1997). The 2015 genome assembly of *S. commersonii* consists of ~ 830 Mb, with 39,290 protein-coding genes, including 126 cold-related genes without orthologs in *S. tuberosum* (Aversano et al. 2015). The heterozygosity in *S. commersonii* reaches 1.5% based on aligning the raw reads to its genome assembly and estimating the heterozygosity by measuring the total number of heterozygous SNP calls over the total number of the callable reads (Aversano et al. 2015). In contrast, the percent heterozygosity in *S. tuberosum* was estimated with only 6373 SNP markers measured against the DM1-3, which resulted in a measure of 53–59% heterozygosity (Hirsch et al. 2013).

*Solanum chacoense* is another closely related tuber-bearing wild species with desirable breeding traits—e.g., disease resistance and resistance to cold-induced sweetening (Leisner et al. 2018). Its high levels of toxic steroidal glycoalkaloids in the tubers, however, are a great disadvantage, and further breeding is required to reduce the glycoalkaloid levels (McCue 2009). The inbred M6 *S. chacoense* clone, developed in 2014 (Jansky et al. 2014), is highly heterozygous and is associated with important agronomic traits like high dry matter, good chip-processing qualities and disease resistance. M6 has also been sequenced and assembled (Leisner et al. 2018), resulting in a genome assembly of ~ 825 Mb, of which 508 Mb has been anchored into 12 pseudomolecules with an estimated 37,740 genes.

In the present study, we carried out comparisons of 12 potato genomes, of which 10 represent native Peruvian landraces, one represents a wild species, and another one represents a native Chilean landrace. The *S. chacoense* M6 clone and *S. commersonii* public genomes (Aversano et al. 2015; Leisner et al. 2018) were included in the study to explore and identify important potential agronomic traits for the future of potato from closely related tuber-bearing potato species. All genomes were compared to the DM1-3 and *S. chacoense* M6 clone to highlight the variation in our 11 landraces and one closely related wild relative genome.

Significant work has previously been done to show CNV—impact on potato (Hardigan et al. 2015). The current study provides further evidence for the importance of CNVs to the potato genome sequence, especially in taxa outside of the Phureja and Stenotomum groups, and in species with varying ploidy levels (2*X*, 3*X*, 4*X* and 5*X*). Moreover, since some of the species analyzed are sexually compatible with the reference genomes and important traits can therefore be transferred to the cultivated potato through introgression, this study is also interesting to breeders and growers. Finally, this is the first report investigating structural variation and polymorphism in potato using more than one reference genome.

## Materials and methods

### Plant materials and sequencing

The germplasm of eleven Peruvian potato accessions and one Chilean accession (TBR) was selected for this study. This consisted of *S. stenotomum* subsp *goniocalyx* Juz. & Bukasov (GON1-CIP 702472 10.18730/9dm*), *S. stenotomum* subsp *goniocalyx* Juz. & Bukasov (GON2-CIP 704393 10.18730/agc$), *S. phureja* Juz. (PHU-CIP 703654 10.18730/9w7j), *S. xajanhuiri* (AJH-CIP 703810 10.18730/a0j9), *Solanum stenotomum* subsp. *stenotomum* Juz. & Bukasov (STN-CIP 705834 10.18730/btda), *S. bukasovii* (BUK-CIP 761748 10.18730/e3ac), *S. tuberosum* subsp. *andigena* Juz. & Bukasov (ADG1-CIP 700921 10.18730/91rp), *S. tuberosum* subsp*. andigena* Juz. & Bukasov (ADG2-CIP 702853 10.18730/9gb8), *Solanum curtilobum* (CUR-CIP 702937 10.18730/9h1y), *S. tuberosum* subsp. *tuberosum* L. (TBR-CIP 705053 10.18730/b3mn), *S. xjuzepczukii* Bukasov (JUZ-CIP 706050 10.18730/c09d) and *S. xchaucha* Juz. & Bukasov (CHA-CIP 707129 10.18730/cs5*), and all are part of the in vitro potato germplasm collection at the International Potato Center (CIP) in Lima, Peru. Genomic DNA was extracted from the leaves of the in vitro plants using EZNA Plant DNA Kit (Omega Bio-Tek, Inc.), following the manufacturer’s instructions. The DNA quality assessment was followed by library preparation and DNA sequencing by Novogene™ Corporation (Beijing, China). Genomic DNA libraries were prepared using the TruSeq Library Construction Kit (Illumina, Inc.) following the manufacturer’s instructions. After the libraries were size selected and purified, they were sequenced using an Illumina HiSeq sequencer (Illumina, Inc.) in paired-end mode (2 × 150 bp). The genomes of GON1 and ADG1 were also sequenced with PacBio’s Single Molecule RS II system technology (https://www.pacb.com/) and with 10*X* Genomics’ GemCode technology (https://www.10xgenomics.com/) by Novogene™. The Illumina paired-end DNA sequencing reads of *S. commersonii* (COM) were obtained from NCBI Sequence Read Archive (SRA) with the SRP050408 identifier and the Illumina paired-end reads for *S. chacoense* (M6) with the SRP097632 identifier. The data are available in NCBI, under the BioProject PRJNA556263; the SRA accessions for the diploid genomes are SRR10244436–SRR10244441, and those for the polyploid genomes are SRR10248510–SRR10248515.

### Alignment against the potato reference genome

The two publicly available potato reference genomes DM1-3 (PGSC 2011) and M6 (Leisner et al. 2018) were used for the detection of copy number variation events (both deletions and duplications) across the 12 accessions. Version 4.04 of the DM1-3 and the v4.1 of M6 reference genomes were retrieved from SpudDB—Potato Genomics Resource database (http://solanaceae.plantbiology.msu.edu/). The pseudomolecules were indexed using BWA MEM v 0.7.17 (Li 2013). The sequencing reads were trimmed using Trimmomatic v0.36 (Bolger et al. 2014) using the following parameters: TruSeq3-PE.fa:2:30:10 LEADING:20 TRAILING:20 SLIDINGWINDOW:5:20 MINLEN:50. The resulting alignments were manipulated using SAMTOOLS v1.9 (Li et al. 2009). Duplicates were marked using Picard v 2.18.9 (Broad-Institute, 2019), and only the properly oriented reads were kept for the structural variation (SV) analyses.

### Determining the portion of whole-genome heterozygosity

Trimmed Illumina sequencing reads were used for the calculation of the percentage of heterozygosity in the genomes. For this, jellyfish v2.2.10 (Marçais and Kingsford 2011) was first used to compute the histogram of the k-mer frequencies. The final k-mer count histogram per genome was used within the GenomeScope 2.0 online platform (Vurture et al. 2017).

### Single nucleotide polymorphism (SNP) analysis

SNPs were detected/called from the processed alignments using Freebayes v1.2.0-2 (Garrison and Marth 2012) with the following criteria: requiring minimum 4*x* coverage in diploids, 6*x* coverage in the triploids, 8*x* coverage in the tetraploids and 10*x* coverage in the pentaploid genomes. Furthermore, SNPs with mapping quality < 20, MQM < 20, MQMR < 20 and SAF && SAR < 0 were removed. The SNPs were annotated with the snpEff tool (Cingolani et al. 2012).

### Copy number variation (CNV) analysis

Genome-wide CNVs were calculated by comparison of median read coverage in 100-bp windows using CNVnator v0.3.3 (Abyzov et al. 2011). The resulting raw CNV calls were filtered in order to keep only the SVs larger than 1000 bp, with a cutoff *p* value of 0.01 and only reads with *q*_0_ quality < 0.5. Significant CNVs were annotated with intansv v1.12.0 (Yao 2018) package in R v3.3.3 (R-Core-Team 2018), using the GFF file with the annotation of the DM1-3 and M6 reference genomes, respectively, to identify which genes were affected by deletions and duplications.

### Significantly enriched gene clusters

Genes with 50% or more of the gene body affected by CNVs were compared to the DM1-3 and M6 reference genomes. CNV gene-enriched clusters were identified by dividing the two reference genomes into overlapping 200-kb bins with an intermediate step size of 10 kb (Hardigan et al. 2016). The number of genes affected by CNVs was calculated in each bin using overlapping bins produced by BEDTOOLS v2.26.0 (Quinlan and Hall 2010). Significant bins were determined using a minimum threshold based on the mean of all genomic windows with addition to three standard deviations (Hardigan et al. 2016). The clusters with the highest number of genes affected by CNVs were further analyzed.

### Principal component analysis of CNV status

CNV-affected genes as defined above were used for clustering analysis. A tertiary matrix with 39,028 genes compared to the DM1-3 was generated along with the genes affected and not affected by CNVs in each of the twelve genomes (3 for duplications, 2 for deletions and 1 for non-CNV-impacted genes). A principal component analysis (PCA) plot was generated using R (R-Core-Team 2018), based on Euclidean distance. Additionally, based on the CNV status of the genes in each of the genomes, two phylogenetic trees were built using PHYLIP v.3.695 (Felsenstein 2005) using the PARS algorithm, which accepts multi-state input.

## Results

### Alignment of 12 potato landrace and wild genomes against two reference genomes shows greater overall match with DM1-3 than with M6

To detect structural variation in the genomes of potato landraces from the GenBank at the International Potato Center (CIP, Lima Peru), genomic DNA was sequenced from a panel of 12 accessions. These accessions were chosen to include representative individuals from each of the seven species, nine taxa and one wild relative proposed by Hawkes (1990). Six are diploids: *Solanum stenotomum* subsp. *goniocalyx* (GON1), *S. stenotomum* subsp. *goniocalyx* (GON2), *S. phureja* (PHU), *S. xajanhuiri* (AJH), *S. stenotomum* subsp. *stenotomum* (STN) and *S. bukasovii* (BUK); two triploids: *S. juzepczukii* (JUZ) and *S. chaucha* (CHA); three tetraploids: *S. tuberosum* subsp. *andigenum* (ADG1), *S. tuberosum* subsp. *andigenum* (ADG2) and *S. tuberosum* subsp. *tuberosum* (TBR); and one pentaploid: *S. curtilobum* (CUR). The genomic DNA reads from the twelve genomes were aligned against the DM1-3 potato reference genome v.4.04 (Hardigan et al. 2016) and against the pseudomolecules of the *S. chacoense* M6 potato reference genome (Leisner et al. 2018). DNA reads from *S. chacoense* (M6) and *S. commersonii* (retrieved from NCBI SRA: SRP097632 and SRP050408, respectively) were also aligned against the DM1-3, and DNA reads from *S. commersonii* (Aversano et al. 2015) were aligned against M6. The *S. commersonii* genome was not used as a reference as the scaffolds were not long enough. Unaligned, unpaired reads and aligned positions with low-quality scores were removed.

**Fig. 1:**
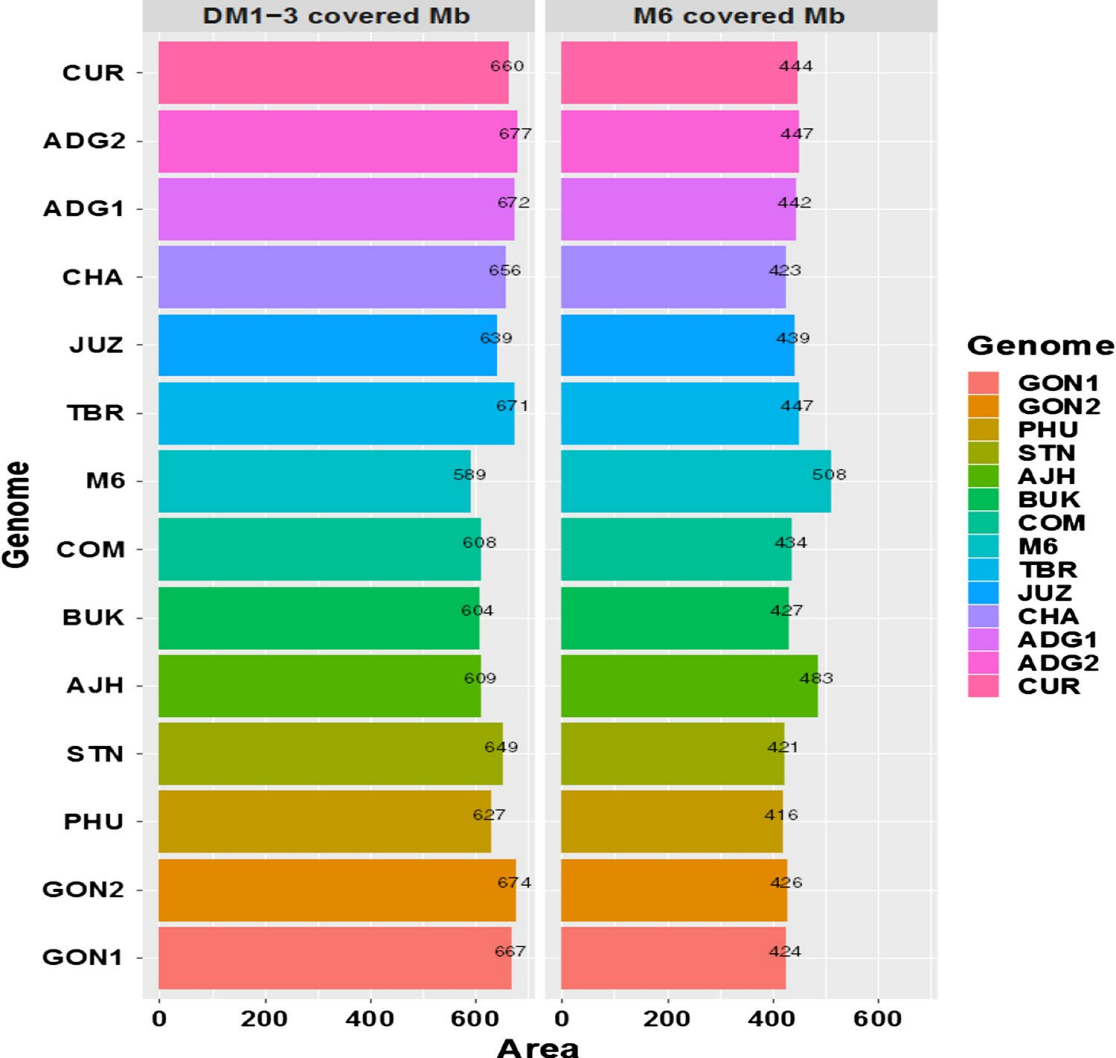
Total amount of the reference genomes: DM1-3 (left) and M6 (right) covered by the aligned reads of 14 potato genomes. The genomes of 12 potato landraces were sequenced, and the reads were aligned against the pseudomolecules of two potato reference genomes, DM1-3 (884 Mb) (Hardigan et al. 2016) and M6 (508 Mb) (Leisner et al. 2018), to show the coverage of each. The sequence reads from the published *Solanum commersonii* (Aversano et al. 2015) were also used in the analysis. GON1—*S. stenotomum* subsp *goniocalyx*; GON2—*S. stenotomum* subsp *goniocalyx*; PHU—*S. phureja*; AJH—*S. xajanhuiri;* STN—*S. stenotomum* subsp. *stenotomum;* BUK—*S. bukasovii;* ADG1—*S. tuberosum* subsp. *andigena*; ADG2—*S. tuberosum* subsp. *andigena*; CUR—*S. curtilobum*; TBR—*S. tuberosum* subsp. *tuberosum*; JUZ—*S. juzepczukii*; CHA—*S. chaucha*; COM—*S. commersonii; and* M6—*S. chacoense*

As shown in Fig. 1, overall, more reads of each genome aligned with DM1-3 than with M6, likely because for the M6 analysis only the pseudomolecules were used as reference. The average size of each reference genome that was covered by the aligned reads was 643 Mb and 436 Mb for the DM1-3 and M6 genomes, respectively. The average read depths for each genome ranged from 35.6*X* (in BUK) up to 50.3*X* (in GON2). The percentage of the reference genome covered by each of the newly sequenced genomes is shown in Fig. 1. The panel of 12 sequenced genomes covered a minimum of 604 Mb and 416 Mb of the DM1-3 and the M6 reference genomes, respectively. Within the 604 Mb of the DM1-3 genome covered, there are 37,395 genes (97% of the total number of genes). Looking at how much of the genomes in our panel align *in common* to each of the two reference genomes, the results show a size of 328 Mb of the diploids and 285 Mb of the polyploids when aligned to DM1-3 and 119 Mb of the diploids and 107 Mb of the polyploids when aligned to M6.

The genome alignments against DM1-3 and M6 were used for the identification of sequence-level variations such as single nucleotide polymorphisms (SNPs) and structural variations such as copy number variation (CNV). High levels of CNVs were observed in the 12 sequenced genomes. Some of the regions of CNVs are identical and, thus, conserved among these genomes. The comparison of the diploids to the DM1-3 showed that in the majority of the diploids (with AJH and BUK and the publicly available COM and M6 genomes being the exceptions), the number of genes impacted by deletions is greater than the number of genes impacted by duplications (Supplementary Figure 1A). Interestingly, in AJH, BUK, COM and M6 the number of deletions is greater than the duplications, but the duplications are larger and thus impact a higher number of genes. Additionally, the polyploids also have fewer, but larger duplications resulting in more genes impacted by duplications than by deletions (Supplementary Figure 1A). Furthermore, the comparison of the diploids and the polyploids with the M6 showed that the number of deletions and duplications is similar in number, but the duplications are again found to be larger, resulting in more genes impacted by duplications (Supplementary Figure 1B). Not unexpectedly, the number of genes impacted by duplications is greater in the polyploids than in the diploids. In general, both reference genome comparisons show that the majority of the deletions occur in the intergenic regions, and thus, duplications affect more genes than the deletions (CNVs were more common in the intergenic regions). Finally, there are many more SNPs in the 12 genomes compared to the DM1-3 than compared with the M6, probably because a smaller portion of the M6 genome was available for alignment. Overall, 275 CNV-impacted genes were in common across the panel of 12 sequenced genomes. Out of those, 109 and 166 genes are impacted by duplication and deletion, respectively.

The average size of the genomic regions impacted by CNVs in the diploids is approximately 311 Mb and 314 Mb compared to DM1-3 and M6, respectively. AJH and BUK have the largest CNV-impacted genome region when compared to DM1-3; however, when compared to M6, it is AJH and PHU that have the two largest CNV-impacted regions. For the polyploid genomes, an average of 378 Mb and 333 Mb of CNV-impacted regions is observed when compared to DM1-3 and M6, respectively. JUZ has the largest CNV-impacted region when compared to DM1-3, followed by CUR. When compared to M6, CUR has the largest CNV-impacted region, followed by JUZ.

The heterozygosity of each of the genomes was estimated in percent using the trimmed Illumina reads. As shown in Table 1, the heterozygosity of the diploids ranges between 1.73% (in GON2) and 4.48% (in AJH). The heterozygosity of the polyploids ranges between 3.52% (in ADG1) and 12.02% (in CUR) (Table 1). This indicates that the higher the ploidy, the higher the heterozygosity and that the heterozygosity is greater outside the Stenotomum and Phureja potato groups.

**Table 1:** Potato genomes sequenced for this study. The table shows their ploidy level and the number of SNPs identified when they were compared to the two reference genomes

Genome	Ploidy	SNPs VS DM1-3	1 Variant per *x* bases	SNPs VS M6	1 Variant per *x* bases	% Heterozygosity
GON1	2*x*	4,452,845	133	4,259,520	95	1.75
GON2	2*x*	4,637,259	126	4,960,736	80	1.73
PHU	2*x*	3,885,936	152	3,862,547	104	1.84
STN	2*x*	5,366,637	110	4,607,143	88	2.06
AJH	2*x*	6,738,160	87	5,503,098	73	4.48
BUK	2*x*	6,962,470	83	5,695,484	71	3.06
ADG1	4*x*	10,488,244	49	7,978,402	50	3.52
ADG2	4*x*	9,998,123	52	7,763,459	51	7.75
TBR	4*x*	9,089,933	58	7,188,156	56	8.43
JUZ	3*x*	10,584,983	48	8,631,219	46	7.3
CHA	3*x*	6,614,894	83	5,350,001	76	3.7
CUR	5*x*	12,968,439	37	8,873,871	45	12.02

### Distribution of single nucleotide polymorphisms detected in the genomes compared to the DM1-3 and M6 reference genomes

The number of SNPs detected compared to the DM1-3 genome ranges from 3.8 million in diploid PHU to 12.9 million in the pentaploid CUR genome (Table 1). The largest number of SNPs detected in the diploids is found in BUK—a wild potato genome—with ~ 7 million SNPs. In the triploids, 6.6 million SNPs are detected in CHA and 10.5 million in JUZ, while the number of SNPs detected in the tetraploids ranges between 7.9 million in ADG1 (7.7 million in ADG2) to 7.1 million in TBR. Moreover, the comparison with M6 demonstrates that the number of SNPs varies between 3.8 million in the diploid PHU up to 8.8 million in the pentaploid CUR. The largest number of SNPs identified in the diploids compared to M6 is 5.6 million (in BUK), in the triploids 8.6 million (in JUZ) and finally in the tetraploids 7.9 million (in ADG1). In summary, the number of SNPs varies between 3.8 million and 10.5 million when compared with DM1-3 and between 3.8 million and 8.6 million when compared with M6 (Table 1).

A total of 96,690 and 373,932 small polymorphisms (SNPs and indels) are found in common between the panel of the 12 genomes: diploids and the polyploids, respectively, while 32,959 are shared among all the ploidy levels. From these, about 65% are in the conserved genome, which is not impacted by any CNVs, and the rest of them in the CNV-impacted genome.

**Fig. 2:**
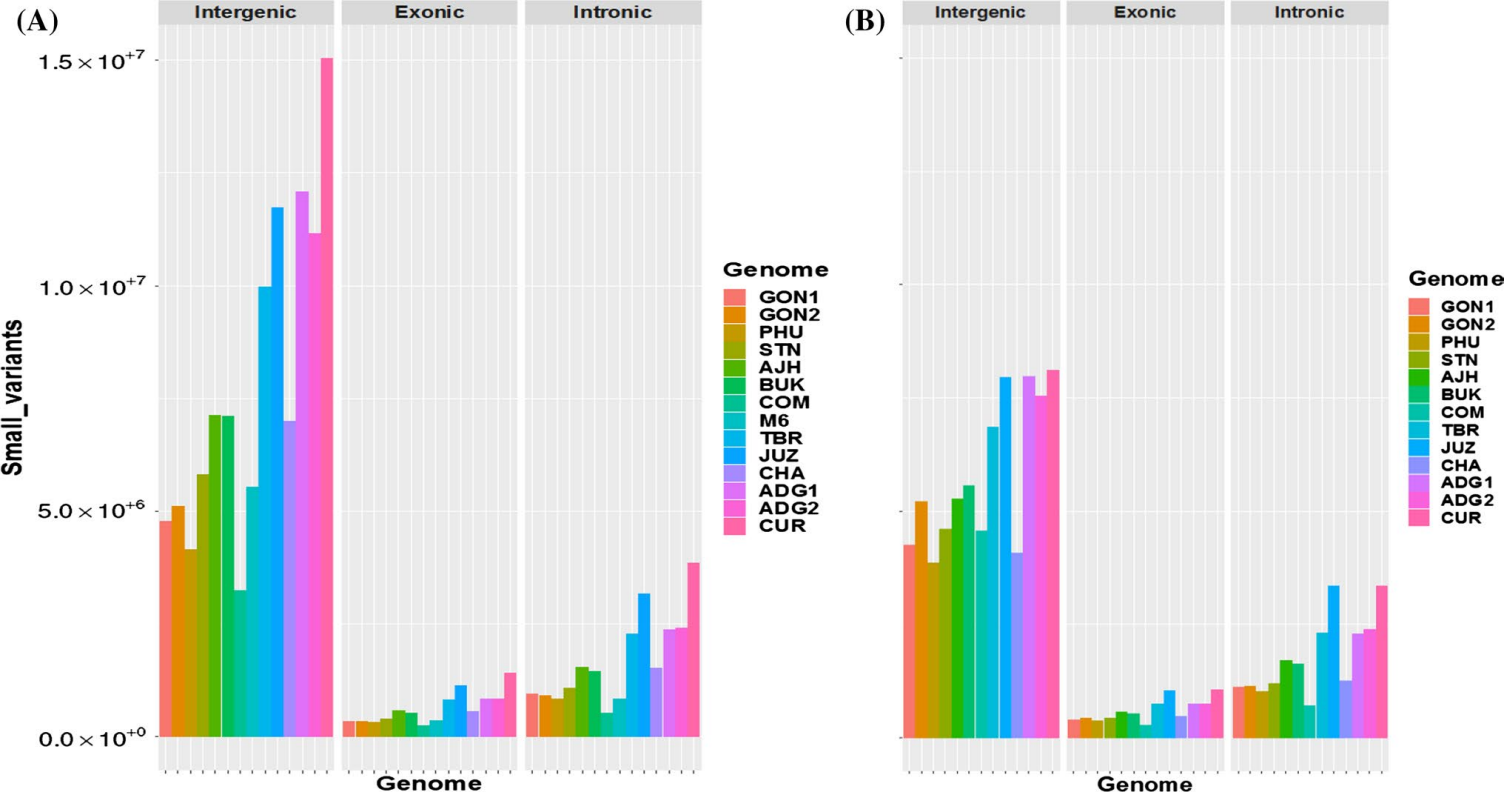
Summary of the total number of small variants (SNPs, indels) identified in 13 potato genomes in intergenic, exonic and intronic regions compared to the **a** DM1-3 and **b** M6 reference genomes. Overall, more SNPs are present in the intergenic regions of the landrace genomes compared with the both reference genomes (DM1-3 on the left and M6 on the right of the figure). Not surprisingly, there are fewest SNPs in exonic regions, and most SNPs are found in the intergenic regions

The identified SNPs were annotated with snpEff (Cingolani et al. 2012), and Fig. 2 shows the total number of small structural variations (SNPs, indels) in the intergenic, exonic and intronic regions, respectively. Based on the results of both reference genome comparisons, the majority of the SNPs are found in the intergenic regions representing 44% of the SNPs (about 22% upstream and 22% downstream). About 51% and 48% of the SNPs consist of missense and silent mutations, respectively, while the remaining 2% are nonsense mutations. The number of indels is smaller than the number of SNPs, with a larger amount of smaller deletions than small insertions in both comparisons.

To identify the most heterozygous regions, biallelic loci were identified in the diploid genomes. Sites that had one or more alternate alleles compared to the reference genome were counted as heterozygous sites. The heterozygosity in the genomes is not spread evenly over the genomes, and some chromosomes are more heterozygous than others based on alternate allele frequency (Supplementary Table 1). The most heterozygous regions in the M6 genome compared to the DM1-3 are found on chromosomes 4, 8 and 9 (Leisner et al. 2018), which was also found in our analysis. This confirms the validity of the pipeline used in the present study (assaying a total of 589 Mb in contrast to the 298 Mb that was previously used). When the landrace genomes are compared to DM1-3, most heterozygous regions are found on chromosomes 1 (an average of ~ 11% heterozygous SNPs) (not in M6) and 4 (an average of ~ 10% heterozygous SNPs), even though some genomes also contained heterozygous regions on chromosomes 3, 6, 8, 9, 10 and 12 (Supplementary Table 1). Specifically, GON1, GON2 and PHU are highly heterozygous in chromosome 9 and AJH and M6 in chromosome 4. Chromosome 1 was the most heterozygous for the polyploids.

The same approach was also used for the identification of the highly heterozygous regions in the genomes compared to the M6 genome. Chromosomes 1 and 12 are consistently the most heterozygous for all the genomes regardless of ploidy level (Supplementary Table 1). Additionally, GON1, GON2, PHU and CHA are highly heterozygous in chromosome 6, while AJH, ADG1, TBR and CUR in chromosome 5, BUK and JUZ in chromosome 3, STN and COM in chromosome 11 and, finally, ADG2 in chromosome 7 (Supplementary Table 1). The highly heterozygous SNPs (compared to both reference genomes) are found predominantly in the intergenic regions based on the annotation by snpEff (Cingolani et al. 2012).

The majority of the SNPs identified across both the diploid and polyploid genomes against both reference genomes are biallelic, with the largest proportion in the ADG1 and CUR genomes (98%). Moreover, most of the biallelic SNPs are of type B (biallelic sites with at least one reference allele and at least one alternate allele). Type B constitutes up to 97% of the biallelic alleles in the ADG1 and CUR genomes.

### Distribution of structural variations in the landrace genomes compared to the DM1-3 and M6 references shows both polymorphism and synergy

#### Size of the CNVs detected

The length of the CNVs detected in the genomes varies in size compared to both DM1-3 and M6 reference genomes. However, in general, when compared to the M6 genome, the CNVs are larger than those detected against the DM1-3 genome. For the DM1-3, the average median size of the CNVs in the panel for the diploids is 6.4 kb, slightly larger in the polyploids (7.7 kb), and for all genomes (all ploidy levels) the median CNV size is 7 kb (Supplementary Table 2). The comparison against the M6 follows a similar pattern, although the size of the CNVs is much larger with an average median CNV length 12.5 kb and 13.5 kb for the diploids and polyploids, respectively (Supplementary Table 3).

Duplications are generally larger than deletions for both diploids and polyploids compared against both reference genomes. However, the largest CNVs detected in the genomes compared to DM1-3 are deletions, even though in general the duplications tended to be larger (Supplementary Table 2). In contrast, when the genomes are compared to M6, the largest CNVs detected are duplications (Supplementary Table 3).

#### Significant gene CNV clusters compared to DM1-3 and M6 reference genomes

To investigate whether large gene clusters were affected with CNVs, the reference genome was split into overlapping bins of 200 kb with a step size of 10 kb, as per (Hardigan et al. 2016). The top three CNV bins identified per genome (Supplementary Table 4, Supplementary Table 5) are not all the same. They involve both duplications and deletions and generally affected disease resistance genes, including those coding for the nucleotide binding site leucine-rich repeat (NBS-LRR) disease resistance proteins. Other CNV-enriched loci contained genes coding for auxin-induced SAURs (small auxin-up RNA), endo-1,4-β-mannosidase and genes of unknown function.

#### Significant gene CNV clusters in the diploids compared to DM1-3

When compared to the DM1-3 reference genome, the CNV-impacted regions in common between the diploid genomes were mostly impacted by deletions (Supplementary Table 6). Genes coding for proteins of unknown function were found across the regions impacted in common by CNVs. Deletions on chromosome 1 affect genes such as methylketone synthase enzyme, involved in the biosynthesis of the methylketones, produced as plant defense against various herbivorous insects by the trichome glands of wild tomato species (Williams et al. 1980; Antonious 2001; Fridman et al. 2005). Additionally, disease resistance genes impacted by deletions are found on chromosomes 4 and 11 (Supplementary Table 6). The region on chromosome 4 contains the *R2* gene, responsible for the resistance against the pathogen *Phytopthora infestance* (Gebhardt and Valkonen 2001). A cluster of genes coding for leucine-rich repeat (NBS-LRR) disease resistance protein, along with others coding for Tobacco mosaic virus (TMV) protein, is impacted by deletions on chromosome 11 (Supplementary Table 6). Finally, genes responsible for biotic and abiotic tolerance are impacted by deletions on chromosomes 9 and 12 (Supplementary Table 6). Some of these genes code for UDP-glycosyltransferase that glycosylate phytohormones and metabolites as a response to biotic and abiotic stresses (Rehman et al. 2018). For instance, they have been shown to play a significant role during TMV infection (Chong et al. 2002; Le Roy et al. 2016) and resistance against Potato Virus Y (PVY) in tobacco (Matros and Mock 2004). On chromosome 12, deletions impact genes coding for important immunity proteins, such as ubiquitin-conjugating enzyme, RNf5, fiber protein Fb34 and others.

#### Significant gene CNV clusters in the diploids compared to M6

Similar to the results from the comparison of the diploid genomes to DM1-3, the chromosomes with CNV-impacted genes in common between all the diploid genomes compared against the M6 genome are chromosomes 1, 4, 9 and 11 (Supplementary Table 6). The majority of these genes are impacted by duplications rather than deletions. Genes involved in stress tolerance are duplicated in chromosomes 1, 4 and 9 (Supplementary Table 6). A gene coding for a major facilitator superfamily (MFS) protein is duplicated in all the diploids when compared to the M6 reference. In Arabidopsis, this protein is responsible for drought tolerance (Remy et al. 2013). Similarly, *DNAJ* genes that were previously found to enhance heat tolerance in transgenic tomatoes (Wang et al. 2019) are duplicated in the diploids, suggesting a possible abiotic tolerance. In pepper, these genes are involved in growth development and are induced by heat stress (Fan et al. 2017). Moreover, genes coding for pentatricopeptide repeat proteins (PPR) are duplicated in the diploid genomes. These were previously shown to have various functions in petunia, including restoring fertility to cytoplasmic male sterility (CMS) lines (Bentolila et al. 2002), and in Arabidopsis, they are involved in salt and drought stress tolerance (Zhu et al. 2012; Lv et al. 2014; Zhu et al. 2014). Duplications in genes coding for serine protease inhibitor (SERPIN) may indicate a defense against insect pests (Jamal et al. 2013). Finally, genes coding for various plant metabolic functions, like 2-oxoglutarate/FE (II)-dependent oxygenase proteins (2OGDs) (Kawai et al. 2014) and others involved in auxin signaling (*SAUR* genes) (Ren and Gray 2015), are duplicated in the diploids compared to M6 (Supplementary Table 6).

#### Significant gene CNV clusters in the polyploids compared to DM1-3

The top CNV-enriched gene clusters in the polyploids also included genes coding for SAURs as well as clusters of genes for tolerance to abiotic stress (Supplementary Table 5). Significant CNV gene clusters in common between the polyploid genomes against the DM1-3 genome were identified (Supplementary Table 7). Interestingly, significant CNV gene clusters in common between the tetraploid genomes were found only on chromosomes 1 and 9 (Supplementary Table 7). In the tetraploid genomes, the regions on chromosome 1 coding for S2 self-incompatibility locus 3.2 protein and F-box protein are duplicated. In addition, on chromosome 1 in all the polyploid genomes, genes coding for male sterility proteins are impacted by duplications compared to DM1-3 (Supplementary Table). Genes coding for heat-shock protein, verticillium wilt resistance protein and TMV resistance protein are also duplicated in the polyploids.

#### Significant gene CNV clusters in the polyploids compared to M6

When compared to the M6 reference genome, all polyploid genomes (ADG1, ADG2, TBR, JUZ, CHA and CUR) have significant CNV-impacted gene clusters on various chromosomes **(**Supplementary Table 5**)**. All regions have more genes impacted by duplications than impacted by deletions. The significant CNV gene clusters in common between the polyploids and the M6 reference genome were for example *SAUR* genes (impacted by duplications on both chromosomes 1 and chromosome 11), genes involved in terpene synthase, C_2_H_2_ and C_2_HC zinc finger proteins, as well as the tetraspanins involved in disease resistance. Proteins involved in vegetative growth and development, such as *gibberellin 3*-*oxidase* genes, are also impacted by duplications, as are genes involved in metabolic processes and response to stimulus (Supplementary Table 7).

#### Significant gene CNV clusters in all the landrace genomes compared to DM1-3

With the exception of the triploid JUZ, all of the genomes, regardless of ploidy levels, have a significantly enriched CNV-impacted gene cluster in the 4.6–4.8-Mb region of chromosome 4 compared to DM1-3 (Supplementary Figure 2). This region contains a disease resistance gene cluster that includes genes that code for the R2 late blight resistance protein, which is implicated in the resistance to *Phytopthora infestans* (Gebhardt and Valkonen 2001). Genes coding for other proteins like EDNR2GH4, EDNR2GH5, EDNR2GH8 and SNKR2GH2 (which are leucine repeat containing proteins) are also detected. In the majority of the genomes, the genes in this region are affected by deletions with an exception in the BUK and M6 genomes, in which the majority of these genes are affected by duplication events.

#### Significant gene CNV clusters in all the landrace genomes compared to M6

Significantly CNV-enriched gene clusters are detected across all the genomes compared to M6 on chromosomes 1 (64.64–64.82 Mb), chromosome 9 (29.23–29.46 Mb) and chromosome 11 (0.88–1.11 Mb) (Supplementary Figure 3). Two of the three regions (those on chromosomes 1 and 11; Supplementary Figure 3A, 3C) contain SAUR gene clusters. The region on chromosome 9 contains 30 genes coding for 2-oxoglutarate (2OG) and Fe(II)-dependent oxygenase superfamily (Supplementary Figure 3B). All the genomes have at least 21 of these genes duplicated, with almost all of them (29) being duplicated in the pentaploid CUR genome.

#### CNV-based classification of 14 potato genomes

**Fig. 3:**
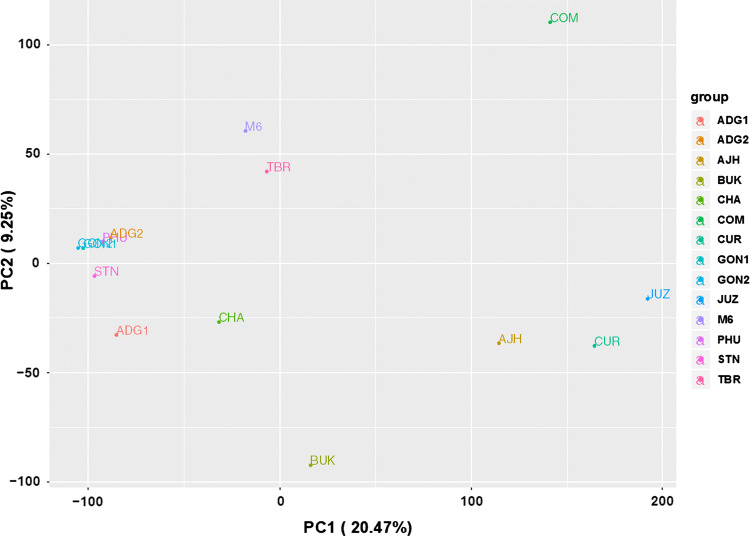
Principal component analysis (PCA) based on the CNV-impacted genes found in the 14 potato genomes compared to the DM1-3 genome, based on Czekanowski genetic distance (also known as Manhattan)

**Fig. 4:**
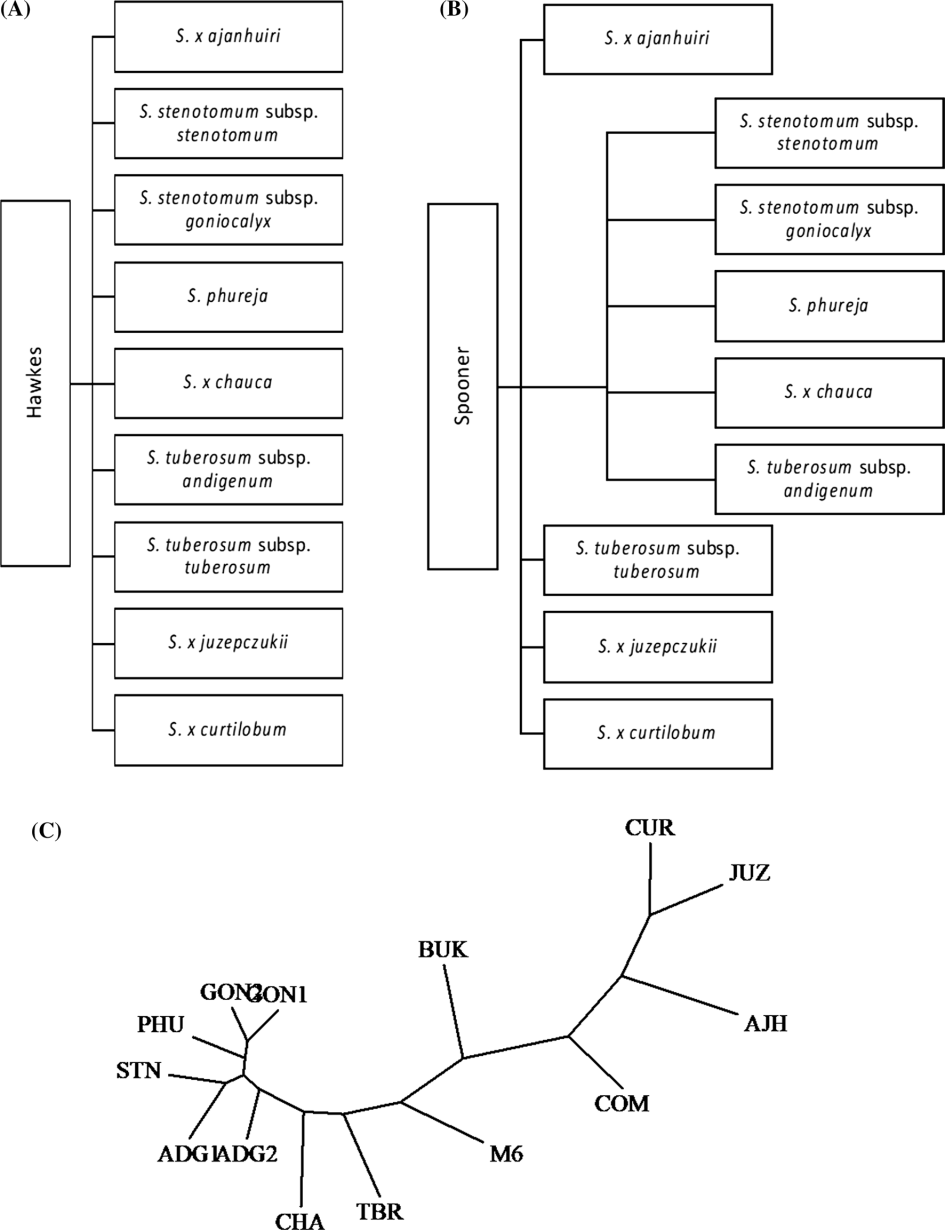
Species taxonomy based on **a** Hawkes (1990) and **b** Spooner et al. (2007) classifications. **c** The phylogenetic tree based on the CNV status of the genes (this study, the same data as used for the PCA). Similar to the PCA plot, CUR, JUZ and AJH genomes cluster closer and they cluster closer to the wild COM genome compared to the other genomes. Moreover, the other wild genome, BUK, is more distant than the other genomes. M6 and TBR genomes are close, while CHA is close to the GON1, GON2, PHU, STN ADG1 and ADG2 cluster

To investigate whether the CNVs have an actual impact on the distance or relatedness of the panel of 12 genomes, M6 and COM, a principal component analysis using the CNV status (duplicated, deleted or non-affected) genes was performed. Figure 3 captures that three clusters and two outliers are apparent: ADG1, ADG2, PHU, GON1, GON2, STN and CHA cluster close together, M6 and TBR make one cluster and AJH, CUR, and JUZ, the bitter potatoes, cluster together, while the two wild species, COM and BUK, are outliers on opposite sides of the graph. Since this largely reflects current taxonomy views, and since a SNP-based phylogenetic analysis was not trivial (because of ploidy and heterozygosity), a phylogenetic analysis was performed with the same CNV-affected gene data as used for the PCA. Figure 4C shows the CNV status-based phylogenetic tree constructed with discrete characters indicating the three statuses of the genes (copy number deleted, duplicated and not impacted). As with the PCA, the GON, PHU, STN and ADG genomes cluster together with CHA close. The BUK and COM are the outliers, yet it is interesting that they map between the bitter genomes (AJH, JUZ, CUR) and the other cultivated taxa.

## Discussion

The results from the current study describe structural variation of 12 potato (*Solanum* sp.) genomes of varying ploidy levels compared with three published reference genomes, DM1-3, M6 and *S. commersonii*.

### Comparison of the analysis with previous studies

Overall, the 12-genome panel matches better with the DM1-3 reference than with the M6 reference genome. The 12 sequenced genomes from this study have more deletions than duplications when compared to the two reference genomes. However, the duplications span larger regions than do the deletions, a fact which was also previously observed in a double monoploid potato panel (Hardigan et al. 2016). The number of deleted genes is greater than the duplicated ones (with the exceptions of AJH, COM, JUZ and CUR) when compared to the DM1-3. This was also found in a previous study of six autotetraploid cultivated potato genomes (Pham et al. 2017). On the contrary, this is not the case when the genomes are compared to the M6 reference, where either the number of duplicated or deleted genes is similar, or duplicated genes are more numerous. In general, the genomes from the wild species (BUK, COM) and bitter cultivated species (AJH, JUZ, CUR) have more genes impacted by duplications than by deletions. The majority of the CNVs impact intergenic regions, and 45–50% of the CNV-impacted genes are of unknown function. The top CNV clusters include genes related to disease resistance, response to stimuli and stress tolerance (heat, frost), which are all important traits in breeding programs.

### Genome comparisons

Potato taxonomy is a topic of active discussion, and the current study makes no claim of authority on that topic. However, of note is that our analyses partially support both major schools of thought (Hawkes and Spooner). In our PCA cluster analysis (Fig. 3), the ADG1, ADG2, PHU, GON1, GON2, STN and CHA clustering supports the view that these genomes can be lumped into a single taxon such as a *S. tuberosum* Andigenum group (Spooner et al. 2007). However, in this cluster CHA could be considered an outlier and could very well be seen as a different species, as has been suggested (Hawkes 1990). The TBR, classified as its own species, is distant from the ADG, PHU, GON, STN and CHA genomes, which is in agreement with previous literature (Hardigan et al. 2017). The TBR is joined by M6 (*S. chacoense*) in an unusual cluster. It was previously reported in wild and cultivated potato that due to unequal wild introgression the genetic distances are smaller between cultivated tetraploids and wild species than between the cultivated tetraploids and their diploid progenitors (Hardigan et al. 2017). This might explain the closeness of TBR and M6. A phylogenetic tree with M6 as an outgroup was also constructed but still placed M6 closest to TBR (data not shown). Also it is observed that the three bitter species, AJH, CUR and JUZ, are clustered together more distantly from the other genomes. BUK and COM, the two wild species, interestingly do not cluster with each other or any other species. COM, however, is closer to the bitter species than to any of the others.

Based on the PCA results, the AJH, CUR and JUZ genomes form one cluster and these are currently thought to be three distinct species by Spooner. It was expected that STN and AJH would be closer together as AJH is considered a hybrid between *S. megistacrolobum* and *S. stenotomum* (Johns et al. 1987) and the cultivated *S. x ajanhuiri* is closer to *S. stenotomum* than to the wild *S x. ajanhuiri* (Johns et al. 1987). However, the AJH accession used in our study clustered with JUZ and CUR, which could be explained by these species being in the bitter potato group and are frost resistant (Johns et al. 1987; Schmiediche et al. 1980). Specifically, JUZ and CUR species are called “papas amargas” or “bitter potatoes” and the bitter taste is due to the high concentrations of particular combination of glycoalkaloids (Schmiediche et al. 1980). The JUZ and CUR species are also resistant to some potato cyst-nematode pathotypes (Dunnett 1957; Christiansen 1977), and CUR is also resistant to bacterial wilt (Martin and French 1977). These genomes have similar characteristics and group together, but do not overlap, which is in agreement with both taxonomic treatments (Hawkes 1990; Spooner et al. 2007).

The wild BUK and COM place at opposite ends in the graph and away from the rest of the genomes. This is not surprising as COM is phylogenetically distant from cultivated potato (Rodríguez and Spooner 2009). BUK, on the other hand, is a potential potato landrace progenitor (Hosaka 1995; Spooner et al. 2005; Hardigan et al. 2015) and thought to be a near relative to the cultivated potato.

TBR and M6 form a cluster in the PCA. Since TBR is a tetraploid, one would have expected it to be closer to the rest of the tetraploids (ADG) along with the other genomes that belong to the Andigenum group. However, it has been previously reported that the genetic distances between the cultivated tetraploids and the wild species are lower than their diploid progenitors due to unequal introgression (Hardigan et al. 2017).

Using two reference genomes instead of one facilitated the CNV analysis of the remarkable genetic diversity of potato. The CNV-based clustering analyses picture this diversity, the relatedness and the uniqueness of these genomes. Specifically, the two public genomes COM and M6 appear in two distinguishable clusters, underlining their differences. They add natural diversity and additional genomic regions, not present in the DM1-3, to the panel, which increases the proof of genetic diversity in this study compared to previous studies on structural variation in potato (Hardigan et al. 2016; Pham et al. 2017).

### A SNP analysis uncovers regions of heterozygosity

The whole-genome sequence analysis using trimmed reads showed that the genomes inside of the Phureja and Stenotomum groups have the lowest level of heterozygosity (Table 1), and our whole-genome SNP analysis unraveled an increasing number of variations and greater heterozygosity with increasing ploidy levels, in agreement with previous studies (Hardigan et al. 2017; Pham et al. 2017). The landrace genomes are highly heterozygous and contain specific regions of higher heterozygosity quite unique to the North American doubled monoploid DM1-3. A non-even distribution of heterozygous regions in potato is supported by previous research (Leisner et al. 2018). Additionally, while around 51% of the SNPs cause missense mutations in both comparisons with DM1-3 and M6, 47% are silent and around 1.2% are nonsense mutations. Similar numbers were previously reported (Pham et al. 2017). Also, in the comparison with the M6 genome, we identified fewer small variations likely due to the fact that the M6 pseudomolecules used in the analysis constitute only 60% of the genome.

A SNP analysis of six autotetraploid potato cultivars (Atlantic, Kalkaska, Missaukee, Russet Norkotah, Snowden and Superior) identified about 8.4 million SNPs compared to the DM1-3 reference genome (Pham et al. 2017). The number of the SNPs identified in the three newly sequenced tetraploid genomes in our study (TBR, ADG1, ADG2) ranged slightly higher, from 9 to 10.4 million SNPs, probably because the six commercial cultivars are inbred, while TBR, ADG1, ADG2 in the present study are landraces and are therefore more likely to be heterozygous, and because a larger region of the genome was used in our analysis. Additionally, the ADG taxa have the greatest admixture (Ellis et al. 2018). Furthermore, the SNPs in our diploid genomes ranged between 3.8 up and 6.9 million in the BUK genome, while a SNP analysis on a doubled monoploid panel had a lower range, from 0.8 up to 4.7 million (Hardigan et al. 2016).

### Several CNV-affected gene clusters are common among potato genomes

In the genomes studied, the number of intergenic CNV events was greater than the intragenic ones. This is consistent with previous CNV studies in other organisms. In the human genome for example, it was shown that CNVs are mostly located outside of gene coding sequences and often affect important regulatory elements (Redon et al. 2006).

Comparing the genomes to both potato reference genomes, in addition to identifying CNVs affecting functionally annotated genes, many CNV-affected genes are hypothetical or conserved hypothetical proteins. This is a common finding based on previous population sequencing studies (Cao et al. 2011; Xu et al. 2012), where it was found that a great number of genes affected by CNVs code for hypothetical or unknown proteins.

### SAUR gene clusters are affected by CNV events in all genomes studied

The most enriched CNV-impacted gene clusters in all genomes compared were those containing auxin-induced SAURs (small auxin-up RNA). These are located on chromosomes 1 (~ 86.97–87.17 Mb), 4 (~ 54.17–54.37 Mb), 6 (~ 56.29–56.49 Mb) and 11 (~ 0.87–1.11 Mb) in the DM1-3 genome and on chromosomes 1 (~ 64.64–64.82 Mb) and 11 (~ 0.88–1.14 Mb) in the M6 genome. In our study, the SAUR genes in comparison with both reference genomes were impacted mostly by duplications (i.e., the SAUR genes are duplicated compared to the SAURs in the reference genomes). The SAURs are a family of auxin-responsive genes that are involved in auxin signaling pathways, regulating a wide range of cellular and developmental processes in plants (Ren and Gray 2015). Various genomic studies have revealed that SAURs are commonly found in clusters or tandem arrays and that there are 134 SAURs in potato, 99 SAURs in tomato, 81 SAURs in *Arabidopsis* and 79 in maize (Hagen and Guilfoyle 2002; Wu et al. 2012; Chen et al. 2014). Interestingly, the study on monoploid potato species also found highly CNV-enriched regions on chromosomes 1 and 11 containing SAURs (Hardigan et al. 2016). A phylogenetic analysis revealed that CNVs play an important role in SAUR gene family expansion in closely related populations of cultivated potato (Hardigan et al. 2016). SAURs are also involved in abiotic stress response, and it has been shown that auxin signaling transduction interacts with other stress signaling pathways in rice (Jain and Khurana 2009).

### Disease resistance gene clusters

Disease resistance genes are another category of genes highly enriched by CNVs compared to both reference genomes. In comparison with DM1-3, all landrace genomes except JUZ have disease resistance genes impacted by deletions on chromosome 4 (~ 4.6–4.8 Mb). This region contains a gene cluster of R2, late blight resistance genes (Li et al. 1998), which was directly affected by deletions. Furthermore, the genomes contained CNVs impacting genes coding for nucleotide binding site leucine-rich repeat (NBS-LRR) disease resistance proteins on chromosomes 8 (~ 47.66–47.86 Mb), 11 (~ 42.72–42. 92 Mb) and 12 (~ 0.6–0.8 Mb). The region on chromosome 11 was previously identified and shown to be impacted by CNVs in a panel of 12 doubled monoploid potato genomes (Hardigan et al. 2016). Regions with disease resistance gene clusters in the 14-genome panel compared to the M6 genome were found on chromosomes 1 (~ 0.39–0.59 Mb), 2 (41.24–41.44 Mb) and 5 (0.2–0.22 Mb). These regions contain NBS-LRR genes. Disease resistance genes are known to be found in clusters in the genomes of many plant species; hence, they are known to undergo rapid evolution as a result of local structural variations (Bergelson et al. 2001) and have been selected during domestication.

### 2-Oxoglutarate/Fe(II)-dependent oxygenase superfamily proteins (2OGDs)

The cluster of genes coding for 2OGD-type proteins was affected by duplication events in the 12-genome panel compared to the M6 reference. The 29.22–29.46-Mb region of chromosome 9 contains a 30-gene cluster coding for 2OGDs. All the potato genomes regardless of ploidy level had at least 11 (GON2, COM) or maximum 21 (JUZ) of these genes impacted by duplications. Proteins in this gene family catalyze various oxidative reactions in plant metabolism, for example DNA repair, biosynthesis of gibberellins (GA), flavonoids, histone demethylation, biosynthesis of plant hormones and various other metabolites (Kawai et al. 2014). Gibberellins are important for many growth and developmental processes in plants, and biosynthesis of GAs includes several 2OGD-dependent reaction steps (Kawai et al. 2014). Flavonoids have diverse functions in plants ranging from plant coloration, protection against UV-B irradiation, nitrogen fixation and adaptation to environmental conditions during periods of abiotic stresses where the biosynthesis of different flavonoid subclasses is catalyzed by various 2OGDs (Farrow and Facchini 2014).

### Genes involved in metabolite biosynthesis

After comparing the 12-genome panel to the M6 reference genome, multiple regions containing genes impacting metabolite biosynthesis were identified as impacted by CNV events. Other highly enriched regions present on chromosomes 1, 3 and 11 contain CNV-affected genes that are involved in terpene synthase, C_2_H_2_ and C_2_HC zinc finger family proteins and tetraspanins, respectively. The plant terpene synthases are responsible for the synthesis of terpene molecules such as isoprenes (tolerance against heat flecks), monoterpenes, sesquiterpenes and diterpenes (Chen et al. 2011). C_2_H_2_ and C_2_HC are the zinc finger domains that are reported to be involved with disease resistance (Emerson and Thomas 2009). Comparative analysis of nine crops revealed zinc finger domains along with NBS-LRR domains in R proteins (Gupta et al. 2012). Tetraspanins are transmembrane proteins that interact with other membrane proteins to form tetraspanin-enriched microdomains, which are involved in various cellular and biological processes that play major roles in pathogenesis and immune response (Wang et al. 2012).

The 32.12–32.37-Mb region of chromosome 3 contains a 35-gene cluster, of which 34 genes are affected by CNVs in *S. bukasovii* and 28 in *S. stenotomum* subsp. *stenotomum*. GO enrichment analysis revealed these genes to be involved in the molecular function “transmembrane transporter activity.” Similarly, enrichment analysis of CNV-affected genes in the 1.24–1.44-Mb region of chromosome 10, revealed genes associated with endoribonuclease activity and protein binding and that the 40.54–40.74-Mb region of chromosome 12 has CNV-affected genes associated with NADH dehydrogenase activity.

## Conclusion

The genomes of a selected set of 12 potato species covering past and current cultivated potato taxa, plus two selected wild species, were studied for structural variation. Similarly to previous studies in other plants, and potato in particular, genes coding for SAUR, methylketones, mannan endo-1,4-β-mannosidase, resistance against *Phytopthora infestans*, NBS-LRR and others of unknown function were found to be impacted by CNVs. However, unlike previous potato studies, we identified other genes, such as those coding for fiber proteins and those involved in self-incompatibility, to be impacted by CNVs in our panel. Genetic diversity through cross-hybridization, polyploidization and speciation makes potato a challenging, but exciting group of species to study. The CNVs represent a source of natural variation that can be tapped for genetic improvement in potato. An important aspect for utilizing CNVs in breeding will be an understanding of the functional impacts of varying copy numbers and an ability to quantify copy numbers with precision and accuracy in high-throughput assays. There is increasing availability of resources for detection of CNVs that will facilitate development of applications for selection and breeding.

This study contains a very diverse genome panel that was not used before for the exploration of CNV in the potato genome. Specifically, a previous comprehensive study of CNV in potato (Hardigan et al. 2016) consisted of significant work in the era, although the panel used was not diverse enough to capture the diversity among different potato taxa. In addition, some of the genomes in the present study are sexually compatible with the cultivated species and so can be used to introduce new desirable traits. Finally, this is the first study in potato exploring CNVs using more than one reference genome. This highlighted the diversity across this panel of potato genomes and identified CNVs in genes implicated in disease resistance and stress tolerance among others.

## Electronic supplementary material

Below is the link to the electronic supplementary material.

Supplementary material 1 (DOCX 289 kb)

Supplementary material 2 (DOCX 50 kb)

## Data Availability

The data reported in this paper have been deposited in the NCBI BioProject database (PRJNA556263).
