# Supplementary material for: Structural genome analysis in cultivated potato taxa
Source: Theor Appl Genet. 2019 Dec 31;133(3):951–66. doi: 10.1007/s00122-019-03519-6 (PMC7021743; doi:10.1007/s00122-019-03519-6)
Supplement: Supplementary file 1 — Supplementary material 1 (DOCX 289 kb) [file 122_2019_3519_MOESM1_ESM.docx]

***Supplementary Figure 1: Duplications and deletions relative to duplicated and deleted genes in 14 potato genomes*** **A.** The number of genes (of which equal or more than 50% of the gene body was) affected by deletions (red) and duplications (blue) across the 14 genomes, along with the total number of deletions (green) and duplications (purple) against the DM1-3 reference genome. In diploids in general, the number of deleted genes was greater than those affected by duplications with AJH and BUK being the exceptions. In contrast, in the polyploid genomes the number of duplicated genes was greater than the deleted ones, with an exception in ADG2 genome. **B.** The number of the genes affected by deletions (red) and duplications (blue) across the 13 genomes (M6 was not analyzed against M6), along with the total number of deletions (green) and duplications (purple) against the M6 reference genome.

Supplementary Figure 1

**A**

**B**

***Supplementary Figure 2: Overview of CNVs over fourteen potato genomes compared with the DM1-3 reference genome, in chromosome 4; 4.6 – 4.8 Mb.*** Distribution of genes of which 50% or more of their gene body was affected by CNVs: with pink the genes impacted by deletions, green with duplications and blue the gene distribution of the DM1-3 in this region.

***Supplementary Figure 3: Overview of CNVs over fourteen potato genomes compared with the M6 reference genome, in chromosomes: A) 1: 64.64 – 64.82 Mb, B) 9: 29.23 – 29.46 Mb and C) 11: 0.88 – 1.11 Mb.*** Distribution of genes of which 50% or more of their gene body was affected by CNVs: with pink the genes impacted by deletions, blue with duplications and green the gene distribution of the DM1-3 in this region.

Supplementary Figure 3 A

Supplementary Figure 3 B

Supplementary Figure 3 C
