# Supplementary material for: Structural genome analysis in cultivated potato taxa
Source: Theor Appl Genet. 2019 Dec 31;133(3):951–66. doi: 10.1007/s00122-019-03519-6 (PMC7021743; doi:10.1007/s00122-019-03519-6)
Supplement: Supplementary file 2 — Supplementary material 2 (DOCX 50 kb) [file 122_2019_3519_MOESM2_ESM.docx]

***Supplementary Table 1: The most heterozygous chromosomes of the genomes when compared to the DM1-3 and M6 genomes.***

| **Genome** | **Top 3 Heterozygous chromosomes VS DM1-3** | **Top 3 Heterozygous chromosomes VS M6** |
| --- | --- | --- |
| **GON1** | Chr04, Chr01, Chr10 | Chr01, Chr12, Chr06 |
| **GON2** | Chr01, Chr09, Chr07 | Chr01, Chr12, Chr06 |
| **PHU** | Chr01, Chr04, Chr06 | Chr01, Chr12, Chr06 |
| **STN** | Chr01, Chr04, Chr06 | Chr12, Chr01, Chr11 |
| **AJH** | Chr04, Chr09, Chr01 | Chr12, Chr01, Chr05 |
| **BUK** | Chr01, Chr03, Chr04 | Chr12, Chr01, Chr03 |
| **COM** | Chr01, Chr04, Chr10 | Chr01, Chr12, Chr11 |
| **M6** | Chr09, Chr04, Chr08 | --------------------- |
| **ADG1** | Chr01, Chr05, Chr10 | Chr12, Chr01, Chr05 |
| **ADG2** | Chr01, Chr04, Chr10 | Chr01, Chr12, Chr07 |
| **TBR** | Chr01, Chr07, Chr09 | Chr01, Chr12, Chr05 |
| **JUZ** | Chr01, Chr04, Chr03 | Chr01, Chr12, Chr03 |
| **CHA** | Chr01, Chr12, Chr04 | Chr12, Chr01, Chr06 |
| **CUR** | Chr01, Chr04, Chr09 | Chr01, Chr12, Chr05 |

***Supplementary Table 2: Summary of the CNVs (deletions and duplications) detected in the A) diploids and B) polyploid genomes against the DM1-3 genome, using CNVnator.***

**A)**

|  | **GON1** | **GON2** | **STN** | **PHU** | **AJH** | **BUK** | **COM** | **M6** |
| --- | --- | --- | --- | --- | --- | --- | --- | --- |
| **Total CNVs** | **20,404** | **19,543** | **22,200** | **21,431** | **22,675** | **23,406** | **25,302** | **19,059** |
| Total deletions | 16,331 | 15,914 | 16,322 | 16,221 | 11,356 | 14,322 | 12,705 | 12,153 |
| Total duplications | 4,073 | 3,629 | 5,878 | 5,210 | 11,319 | 9,084 | 12,997 | 6,906 |
| **Genic CNVs (%)** | **20.2%** | **17.8%** | **26.7%** | **25%** | **59.6 %** | **45.9%** | **71.2%** | **33.7%** |
| **Mean CNV length** | **10.5 kb** | **10.1 kb** | **12.2 kb** | **11.6 kb** | **18.4 kb** | **15.8 kb** | **21.1 kb** | **15.5 kb** |
| **Median CNV length** | **4 kb** | **3.7 kb** | **5 kb** | **4.9 kb** | **10.2 kb** | **7.2 kb** | **9.8 kb** | **6.4 kb** |
| Median deletion length | 3.6 kb | 3.3 kb | 4.2 kb | 4.1 kb | 6.1 kb | 5.1 kb | 6.2 kb | 4.4 kb |
| Median duplication length | 5.5 kb | 4.8 kb | 7.3 kb | 7.4 kb | 15.7 kb | 11.2 kb | 14.3 kb | 10.8 kb |
| **Total large CNVs*** | **160** | **144** | **333** | **187** | **283** | **345** | **440** | **344** |
| **Size of the largest CNV** | **515.2 kb** | **730.1 kb** | **506.8 kb** | **629.9 kb** | **529.5 kb** | **582.5 kb** | **621.2 kb** | **501.1 kb** |
| Genes affected by deletions | 5,202 | 4,782 | 6,043 | 5,616 | 4,575 | 6,431 | 6,690 | 5,028 |
| Genes affected by duplications | 2,684 | 2,175 | 4,385 | 4,134 | 18,711 | 11,488 | 21,099 | 8,115 |
| **Total CNV-affected genes^$^** | **7,886** | **6,957** | **10,428** | **9,750** | **23,286** | **17,919** | **27,789** | **13,143** |

|  | **ADG1** | **ADG2** | **TBR** | **JUZ** | **CHA** | **CUR** |
| --- | --- | --- | --- | --- | --- | --- |
| **Total CNVs** | **21,489** | **18,243** | **21,323** | **22,875** | **21,790** | **22,694** |
| Total deletions | 15,243 | 12,105 | 13,719 | 10,091 | 13,452 | 10,900 |
| Total duplications | 6,246 | 6,138 | 7,604 | 12,784 | 8,338 | 11,794 |
| **Genic CNVs (%)** | **26.8%** | **22.4%** | **37.5%** | **77.8%** | **35.3%** | **69%** |
| **Mean CNV length** | **11.8 kb** | **12.1 kb** | **14.3 kb** | **21.5 kb** | **15.2 kb** | **19.2 kb** |
| **Median CNV length** | **4.7 kb** | **5.1 kb** | **6.7 kb** | **11.6 kb** | **7.6 kb** | **10.4 kb** |
| Median deletion length | 3.8 kb | 3.7 kb | 4.9 kb | 6.4 kb | 5.2 kb | 6 kb |
| Median duplication length | 7.3 kb | 8.1 kb | 10.8 kb | 17.4 kb | 12.3 kb | 16.1 kb |
| **Total large CNVs*** | **195** | **148** | **221** | **465** | **226** | **317** |
| **Size of the largest CNV** | **900.3 kb** | **670.8 kb** | **1.1 Mb** | **594 kb** | **900.3 kb** | **646.8 kb** |
| Genes affected by deletions | 5,003 | 3,741 | 5,105 | 3,972 | 5,095 | 4,238 |
| Genes affected by duplications | 5,453 | 5,012 | 9,537 | 26,411 | 8,681 | 22,684 |
| **Total CNV-affected genes^$^** | **10,456** | **8,753** | **14,642** | **30,383** | **13,776** | **26,922** |

**2B)**

** Large CNVs are defined as having a length > 100 kb. The length of the CNVs found in ST4.03ch00 are not counted in the length metrics, only the impacted genes are counted in the table.*

*$ This total includes genes that are affected by both deletions and duplications*

***Supplementary Table 3: Summary of the CNVs (deletions and duplications) detected in the A) diploids and B) polyploid genomes against the M6***

|  | **GON1** | **GON2** | **STN** | **PHU** | **AJH** | **BUK** | **COM** |
| --- | --- | --- | --- | --- | --- | --- | --- |
| **Total CNVs** | **16,679** | **16,378** | **17,471** | **16,713** | **6,889** | **9,617** | **17,096** |
| Total deletions | 7,956 | 8,227 | 8,458 | 7,667 | 2,740 | 4,886 | 8.908 |
| Total duplications | 8,723 | 8,151 | 9,013 | 9,046 | 4,149 | 4,731 | 8,188 |
| **Genic CNVs (%)** | **50.5%** | **43.7%** | **51.2%** | **54.2%** | **59%** | **46%** | **38.3%** |
| **Mean CNV length** | **19.1 kb** | **17.7 kb** | **18.5 kb** | **20 kb** | **49.7 kb** | **30 kb** | **16.8 kb** |
| **Median CNV length** | **10 kb** | **9 kb** | **9.8 kb** | **10.9 kb** | **23.6 kb** | **15.6 kb** | **8.7 kb** |
| Median deletion length | 5.7 kb | 5.2 kb | 5.4 kb | 5.9 kb | 9.4 kb | 8.4 kb | 5.7 kb |
| Median duplication length | 16.5 kb | 15.5 kb | 16.3 kb | 17.3 kb | 45.4 kb | 28.6 kb | 13.9 kb |
| **Total large CNVs*** | **328** | **283** | **308** | **373** | **948** | **546** | **245** |
| **Size of the largest CNV** | **439 kb** | **584.4 kb** | **568.6 kb** | **434.9 kb** | **745.8 kb** | **1 Mb** | **358 kb** |
| Genes affected by deletions | 2,335 | 2,085 | 2,361 | 2,253 | 1,172 | 2,095 | 2,975 |
| Genes affected by duplications | 16,737 | 14,402 | 16,967 | 18,188 | 21,116 | 15,267 | 11,468 |
| **Total CNV-affected genes^$^** | **19,072** | **16,487** | **19,328** | **20,441** | **22,288** | **17,362** | **14,443** |

**A)**

**3B)**

|  | **ADG1** | **ADG2** | **TBR** | **JUZ** | **CHA** | **CUR** |
| --- | --- | --- | --- | --- | --- | --- |
| **Total CNVs** | **16,378** | **11,250** | **8,624** | **12,250** | **14,759** | **11,538** |
| Total deletions | 8,227 | 4,419 | 4,016 | 4,450 | 6,128 | 3,915 |
| Total duplications | 8,151 | 6,831 | 4,608 | 7,800 | 8,631 | 7,623 |
| **Genic CNVs (%)** | **43.7%** | **40.7%** | **48.8%** | **65.4%** | **59.8%** | **69.3%** |
| **Mean CNV length** | **17.3 kb** | **23.3 kb** | **34.7 kb** | **31.9 kb** | **24.5 kb** | **34.8 kb** |
| **Median CNV length** | **8.6 kb** | **10.7 kb** | **17.2 kb** | **14.8 kb** | **12.6 kb** | **16.9 kb** |
| Median deletion length | 5.2 kb | 5.3 kb | 8.8 kb | 6.2 kb | 6.2 kb | 6.4 kb |
| Median duplication length | 14.7 kb | 18 kb | 31.6 kb | 24.4 kb | 20.1 kb | 28.6 kb |
| **Total large CNVs*** | **294** | **437** | **676** | **764** | **547** | **927** |
| **Size of the largest CNV** | **371.8 kb** | **560.8 kb** | **838.6 kb** | **5 Mb** | **505.5 kb** | **511.8 kb** |
| Genes affected by deletions | 2,085 | 1,217 | 1,826 | 1,008 | 1,637 | 901 |
| Genes affected by duplications | 14,402 | 14,173 | 16,594 | 23,665 | 20,923 | 25,262 |
| **Total CNV-affected genes^$^** | **16,487** | **15,390** | **18,420** | **24,673** | **22,560** | **26,163** |

***Supplementary Table 4: Top 3 gene enriched CNV bins in the 8 diploid genomes against the DM1-3 and the M6 reference genomes.***

| Genome | Top 3 CNV bins VS **DM1-3** | Top 3 CNV bins VS **M6** |
| --- | --- | --- |
| **GON1** | - ST4.03ch12**^$^**: mannan – endo – 1,4, - β mannosidase genes (del) - ST4.03ch04: disease resistance genes (del + dup) - ST4.03ch05: receptor kinase, wall associate kinase coding genes etc. (del) | - M6_v4.1ch01**^*^**: Auxin – induced SAUR gene cluster (dup) - M6_v4.1ch05**^$^**: genes coding for proteins of unknown function, NB-ARC coding genes, chaperone subunit etc. (dup) - M6_v4.1ch09: 2 – oxoglutarate genes, bHLH etc. (dup) |
| **GON2** | - ST4.03ch12**^$^**: mannan – endo – 1,4, - β mannosidase genes (del) - ST4.03ch00: genes of various functions i.e matrix metalloprotease coding gene, MYC1 (dup) - ST4.03ch08: disease resistance genes, *R2* (del) | - M6_v4.1ch05: genes coding for flavin – binding proteins, NAD(P), terpenesythase etc. (dup) - M6_v4.1ch07: various genes; including D – mannose binding lectin coding gene, cytochrome P450, etc. (del + dup) - M6_v4.1ch01: Auxin – induced SAUR gene cluster (dup) |
| **PHU** | - ST4.03ch11: disease resistance gene cluster, *R2* (del + dup) - ST4.03ch02: conserved gene cluster of unknown function (dup) - ST4.03ch04: Auxin - induced SAUR gene cluster (dup) | - M6_v4.1ch11**^!^**: Auxin – induced SAUR gene cluster (del + dup) - M6_v4.1ch01**^*^**: Auxin – induced SAUR gene cluster (dup) - M6_v4.1ch05**^$^**: genes coding for hypothetical protein, Rubisco methyltransferase, NB-AC domain containing etc. (dup) |
| **STN** | - ST4.03ch11: disease resistance gene cluster (del) - ST4.03ch04: genes coding for LRR containing proteins, *R2* (del + dup) - ST4.03ch12: genes of various functions (del + dup) | - M6_v4.1ch01**^*^**: Auxin – induced SAUR gene cluster (dup) - M6_v4.1ch07: genes coding for gibberellin 3 – oxidase, pyridoxine biosynthesis, α – β – hydrolases, lectin protein kinase family etc. (dup) - M6_v4.1ch11**^!^**: Auxin induced SAUR gene cluster, F-box etc. (dup) |
| **AJH** | - ST4.03ch02: genes involved in the carbohydrate metabolic process (dup) - ST4.03ch05: flavonol 4’ – sulfotransferase coding gene, late blight resistance etc. (dup) - ST4.03ch06: male sterility MS5, nodulin – 26, auxin regulated coding gene etc. (dup) | - M6_v4.1ch11**^!^**: Auxin – induced SAUR gene cluster (dup) - M6_v4.1ch01**^*^**: Auxin – induced SAUR gene cluster (dup) - M6_v4.1ch01: genes coding for NB-ARC, LRR, flavin – binding kelch repeat, F-box etc. (del) |
| **BUK** | - ST4.03ch03: flavonol synthase/ flavone 3 – hydroxylase coding genesetc (dup) - ST4.03ch04: genes coding for alcohol dehydroxygenase ADH, auxin responsive family protein, lysine/histidine transporter etc. (dup) - ST4.03ch10: non-structural maintenance of chromosome element, conserved gene of unknown function etc. (dup) | - M6_v4.1ch11**^!^**: Auxin – induced SAUR gene cluster (del + dup) - M6_v4.1ch01: genes coding for NB-ARC, hypothetical proteins, bHLH, LRR, F-box etc. (dup) - M6_v4.1ch01**^*^**: Auxin – induced SAUR gene cluster (dup) |
| **COM** | - ST4.03ch01**^*^**: Auxin – induced SAUR gene cluster (dup) - ST4.03ch11: Auxin – induced SAUR gene cluster (dup) - ST4.03ch06: Auxin – induced SAUR gene cluster (dup) | - M6_v4.1ch01: genes coding for NB - ARC domain, LRR, 3 – flavin – binding protein etc. (del) - M6_v4.1ch05**^$^**: genes coding for EXC family protein, Rubisco, disease resistance, LRR etc. (dup) - M6_v4.1ch12: genes coding for bHLH, fatty acid hydroxylase, tetraspanins, knotted-1 like etc. (dup) |
| **M6** | - ST4.03ch01**^*^**: Auxin – induced SAUR gene cluster (dup) - ST4.03ch04: genes coding for lipid binding protein, transducing family protein etc. (dup) - ST4.03ch04: conserved genes of unknown function, ethylene – inducing xylanase, 1,4 – α – glucan branching etc. (dup) | -------------------------------------------------- |

*, $, ! These regions are the same in the genomes where the according symbol is found.

dup – duplication event

del – deletion event

***Supplementary Table 5: Top 3 gene enriched CNV bins in the 6 polyploid genomes against the DM1-3 and the M6 reference genomes.***

| Genome | Top 3 CNV bins VS DM1-3 | Top 3 CNV bins VS M6 |
| --- | --- | --- |
| **ADG1** | - ST4.03ch12: genes coding for extension Ext1, stress – associated proteins etc. (dup) - ST4.03ch05**^*^**: genes coding for late blight resistance protein, retroelement, ribulose – 1.5 bisphosphate, carboxylase/oxygenase etc. (dup) - ST4.03ch12: genes coding for thioredoxin domain – containing protein, fertility restorer etc. (dup) | - M6_v4.1ch01**^&^**: Auxin – induced SAUR gene cluster (dup) - M6_v4.1ch11**^+^**: Auxin – induced SAUR gene cluster, F-box domain coding genes, etc. (del + dup) - M6_v4.1ch02: SINE3-like coding gene, RING/U – box, peroxidase, transferases etc. (dup) |
|  |  |  |
|  |  |  |
| **ADG2** | - ST4.03ch07: geranyl geranyl pyrophosphate synthase, conserved gene of unknown function etc. (dup) - ST4.03ch07: ATNMNAT, RNA – binding protein, zinic transporter etc. (dup) - ST4.03ch03: genes coding for LRR, F-box, TPR domains, genes of unknown functions, pentatricopeptide, etc. (dup) | - M6_v4.1ch05**^+^**: Auxin – induced SAUR gene cluster (dup) - M6_v4.1ch11: Auxin – induced SAUR gene cluster (dup) - M6_v4.1ch01^&^: Auxin – induced SAUR gene cluster (dup) |
|  |  |  |
|  |  |  |
| **TBR** | - ST4.03ch11**^$^**: Auxin – induced SAUR gene cluster (dup) - ST4.03ch05**^*^**: ribulose – 1,5 bisphosphate, carboxylase/oxygenase, conserved genes of unknown function etc. (dup) - ST4.03ch05: signal transducer, nodulin family, glycine – rich cell wall structural protein 1, etc. (dup) | - M6_v4.1ch01^&^: Auxin – induced SAUR gene cluster, PPR, calcium binding EF- hand coding genes etc. (dup) - M6_v4.1ch07: late embryogenesis abundant (LEA) coding gene, methylotransferase family protein, lectin protein kinase, gibberellin 3 -oxidase etc. (dup) - M6_v4.1ch09: genes coding for 2 – oxoglutarate, bHLH, nodulin MtN21/EaMA like transporter family, etc. (dup) |
|  |  |  |
|  |  |  |
| **JUZ** | - ST4.03ch01: Auxin – induced SAUR gene cluster (dup) - ST4.03ch03: conserved genes of unknown function, protein HVA22, etc. (dup) - ST4.03ch05**^*^**: ribulose – 1,5 bisphosphate, carboxylase/oxygenase, conserved genes of unkown function etc. (dup) | - M6_v4.1ch11**^+^**: Auxin – induced SAUR gene cluster, F- box, TPR, oxidoreductase etc. (del + dup) - M6_v4.1ch01^&^: Auxin – induced SAUR gene cluster, calcium – binding, methyl adenosine nucleoside etc. (dup) - M6_v4.1ch05: genes coding for F-box domain containing proteins, LAG1 longevity assurance homolog, ECA1 gametogenesis related, FAD/NAD(P) – binding oxidoreductase family protein, etc. (dup) |
|  |  |  |
| **CHA**    **CUR** | - ST4.03ch05**^*^**: Auxin – induced SAUR gene cluster (dup) - ST4.03ch11**^$^**: Auxin induced SAUR gene cluster (del + dup) - ST4.03ch12: genes coding for zinic finger, CYP82C4, ubiquitin carrier, gens of unknown functions, etc. (dup) | - M6_v4.1ch01**^&^**: Auxin – induced SAUR gene cluster, PPr, CCHC – type zinc finger, etc. (dup) - M6_v4.1ch11**^+^**: Auxin – induced gene cluster (del + dup) - M6_v4.1ch02: genes coding for TPX2 (targeting protein for Xklp2), hydrolases, glucose – 6 – phosphate-dehydrogenase, etc. (dup) |
|  | - ST4.03ch11**^$^**: Auxin – induced SAUR gene cluster(dup) - ST4.03ch06: genes coding for male sterility MS5, nodulin 26, endo 1,4 beta xylanase (xylA), etc. (dup) - ST4.03ch05**^*^**: Auxin – induced SAUR gene cluster (dup) | - M6_v4.1ch11**^+^**: Auxin – induced SAUR gene cluster, F-box coding genes, etc. (dup) |
|  |  |  |

***Supplementary Table 6: Significant CNV gene clusters in common between the diploid genomes when compared to DM1—3 and M6 reference genomes along with the variation status; duplicated or deleted.***

|  | **Compared to the DM1-3** | **Compared to the M6** |
| --- | --- | --- |
| **Chromosome 01** | - **85 – 85.21 Mb – mostly impacted by deletions**   Cluster of 18 genes coding for methylketone synthase enzyme. | - **54.44 – 54.64 Mb – mostly impacted by duplications**   A 55 – gene cluster, including genes coding for Major Facilitator Superfamily (MFS) protein of membrane transport secondary carriers, for argirine-rich cyclin, chitin elicitor receptor kinase, for DNAJ heat shock N terminal domain-containing protein and for the pentatricopeptide repeat (PPR) superfamily protein. |
| **Chromosome 04** | - **4.6 – 4.8 Mb – impacted by deletions (in GON1, GON2, PHU and STN), impacted by duplications (in AJH, BUK, COM and M6)**   Cluster of 32 genes, specifically disease resistance genes, including *R2* gene. | - **35.41 – 35.61 Mb – mostly impacted by deletions**   A cluster of 33 genes coding for serine protease inhibitor (SERPIN), for UDP-Glycotransferase superfamily proteins, MFS proteins and disease resistance ADG1- like proteins.   - **36.47 – 36.67 Mb – mostly impacted by duplications**   A cluster of 32 genes coding for ribonucleases, leucine carboxyl methyltransferases, mannose-binding lectin family protein, PPRs and a NmrA-like negative transcriptional regulator family protein. |
| **Chromosome 09** | - **59.86 – 60.06 Mb – mostly impacted by deletions**   A cluster of 26 genes, including those coding for glycotransferase, cembratrienol synthase, a gene coding for a xyloglucan endotrabskycosylase, a hypothetical salt-inducible protein, another for F-box protein, Cytochrome P450 and other conserved genes of unknown function. | - **29.23 – 29.46 Mb – mostly impacted by duplications**   A cluster of 43 genes; 30 of these code for 2-oxoglutarate (2OG) and FE (II)-dependent oxygenase superfamily protein, in addition to others coding for a nodulin MtN21/EamA-like transporter family protein, an electron transfer flavoprotein, bHLH, and the UDP-glycosyltrasnferase superfamily. |
| **Chromosome 11** | - **1.33 – 1.53 Mb – impacted by deletions (duplications in M6)**   A cluster of 17 genes, including genes coding for Leucine Rich Repeat family proteins, TMV resistance protein N | - **0.89 – 1.11 Mb – mostly impacted by deletions (except AJH)**   A 54 gene cluster, including 32 genes coding for SAUR-like auxin responsive protein family, along with ethylene-responsive element coding genes. |
| **Chromosome 12** | - **0.6 – 0.8 Mb – impacted by deletions (PHU and COM did not show CV)**   A 29 – gene cluster, including 7 mannan endo-1,3-beta-mannosidase 1 coding genes, others coding for important plant immunity proteins, such as Ubiquitin conjugating enzyme, Rnf5 and stress tolerance, like Fiber protein Fb34, metallothionein. | **No significant CNV gene clusters in common between the diploids.** |

***Supplementary Table 7: Significant CNV gene clusters in common between the polyploid genomes when compared to DM1—3 and M6 reference genomes along with the variation status; duplicated or deleted.***

|  | **Compared to the DM1-3** | **Compared to the M6** |
| --- | --- | --- |
| **Chromosome 01** | - **4.4 – 5.38 Mb – duplicated only in the tetraploids, no CNV-impacted genes in other genomes**   A 66-gene cluster of unknown function, others coding for Cytochrome P450, S2 self-incompatibility locus-linked pollen 3.2 protein, mannan endo-1,4-beta-mannosidase 4, F-box family protein and ethylene-responsive TF.   - **75.8 – 85 Mb – impacted by duplications**   Various genes; like *FIONA* coding for FRIGIDA AND Type I MADS box TF, others coding for male sterility protein, low temperature and salt responsive protein, heat shock proteins sugar transporters, verticillium wilt disease resistance protein, glycosyl transferase, auxin response protein, TMV resistance protein N, 2O-Fe (II) oxygenase.   - **86.98 – 87.2 Mb – impacted by duplications but no CNV-impacted genes in ADG1**   A 43-gene cluster, including 17 auxin induced SAUR genes. | - **55.21 – 55.44 Mb – mostly impacted by duplications**   A 41-gene cluster, containing genes coding for terpene synthase.   - **60.4 – 60.6 Mb – mostly impacted by duplications**   A gene cluster of 30 genes involved in the biological process of intercellular sequestration of iron.   - **64.64 – 64.82 Mb – mostly impacted by duplications**   A SAUR-auxin induced coding gene cluster. |
| **Chromosome 03** | **No significant CNV gene clusters in common between the polyploids.** | - **41.32 – 41.53 Mb – mostly impacted by duplications**   A 35-gene cluster of genes coding for tetraspanin8, tetraspanin12 and a calcium-dependent phosphotriesterase protein. |
| **Chromosome 05** | - **0 – 0.21 Mb – mostly impacted by duplications**   A 33 disease resistance gene cluster as well as other genes involved in cell metabolic processes. | **No significant CNV gene clusters in common between the polyploids.** |
| **Chromosome 07** | - **55.36 – 55.57 Mb – duplications in all polyploids but no CNV-impacted genes in ADG1**   A cluster of 186 genes coding for enoyl-CoA hydratase, mitochondrial, zinic transporter, lipid binding protein and a fascilin-like arbinogalactan protein 10. | - **32.12 – 32.37 Mb – mostly impacted by duplications**   A cluster of 43 genes coding for gibberellin 3-oxidase, TPR HCO_3_. |
| **Chromosome 09** | - **59.4 – 61 Mb – deleted in ADG1, ADG2, duplicated only in TBR, no CNV-impacted genes in other genomes**   A 61-gene cluster containing Tospovirus resistance genes. | - **29.2 – 29.46 Mb – mostly impacted by duplications**   A cluster of 43 genes, 30 of which code for 2OGD. |
| **Chromosome 10** | **No significant CNV gene clusters in common between the polyploids.** | - **1.24 – 1.44 Mb – only impacted by duplications**   A cluster of 40 genes involved in various functions, like metabolic processes and response to stimulus. |
| **Chromosome 11** | **No significant CNV gene clusters in common between the polyploids.** | - **0.88 – 1.14 Mb – mostly impacted by duplications**   A SAUR-auxin induced gene cluster.   - **37.86 – 38.08 Mb – mostly impacted by duplications**   A cluster of 44 genes, coding for tetratricopeptide repeats (TPR), C_2_H_2_, C_2_HC zing fingers superfamily proteins. |
| **Chromosome 12** | - **58.12 – 58.34 Mb – impacted by duplications**   A 34 gene cluster involved in cellular metabolic processes. | - **40.54 – 40.74 Mb – mostly impacted by duplications**   A cluster of 28 genes, 18 of which code for hypothetical proteins. |
